# Role of Nrf2 Signaling Cascade in Breast Cancer: Strategies and Treatment

**DOI:** 10.3389/fphar.2022.720076

**Published:** 2022-04-29

**Authors:** Hitesh Kumar, Rachna M. Kumar, Devanjali Bhattacharjee, Preethi Somanna, Vikas Jain

**Affiliations:** Department of Pharmaceutics, JSS College of Pharmacy, JSS Academy of Higher Education and Research, Mysuru, India

**Keywords:** Nrf2, breast cancer, drug resistance, angiogenesis, metastasis

## Abstract

Breast cancer is the second leading cancer among all types of cancers. It accounts for 12% of the total cases of cancers. The complex and heterogeneous nature of breast cancer makes it difficult to treat in advanced stages. The expression of various enzymes and proteins is regulated by several molecular pathways. Oxidative stress plays a vital role in cellular events that are generally regulated by nuclear factor erythroid 2-related factor 2 (Nrf2). The exact mechanism of Nrf2 behind cytoprotective and antioxidative properties is still under investigation. In healthy cells, Nrf2 expression is lower, which maintains antioxidative stress; however, cancerous cells overexpress Nrf2, which is associated with various phenomena, such as the development of drug resistance, angiogenesis, development of cancer stem cells, and metastasis. Aberrant Nrf2 expression diminishes the toxicity and potency of therapeutic anticancer drugs and provides cytoprotection to cancerous cells. In this article, we have discussed the attributes associated with Nrf2 in the development of drug resistance, angiogenesis, cancer stem cell generation, and metastasis in the specific context of breast cancer. We also discussed the therapeutic strategies employed against breast cancer exploiting Nrf2 signaling cascades.

## 1 Introduction

Breast cancer is a deadly disease affecting the majority of the female population. In 2020, approximately 2.26 million cases were recorded globally, and 6,85,000 deaths were reported. Out of 2,81,591 reported cases, 48,407 deaths were observed in the United States, while with a similar number (2,54,881) of cases in India, 1,24,975 deaths were reported (https://www.who.int/cancer/country-profiles/en/). According to GLOBOCAN (2020), 2.2 million cases were reported, and it is the second most prevalent cancer among women. Breast cancer is diagnosed in one in four women globally (Morphology). According to immunohistochemical markers, breast cancer has five subtypes that differ in prognosis and therapeutic targets: 1) luminal A (ER positive and/or PR positive and HER2 negative), 2) luminal B (estrogen receptor positive and/or progesterone receptor positive and HER2 positive), 3) HER2 overexpressing (estrogen receptor and progesterone receptor negative and HER2 positive), 4) basal like (estrogen receptor/progesterone receptor/HER2 negative, cytokeratin 5/6 positive, and/or epidermal growth factor receptor positive), and 5) normal breast like. TNBC is a type of breast cancer in which estrogen receptor (ER), progesterone receptor (PR), and human epidermal growth factor receptor-2 (HER2) are not expressed. Gene expression in triple-negative breast cancer often classifies it as a subtype of basal-like breast cancer (Kumar and Aggarwal, 2016). Approximately 15–20% of cases represent TNBC, which has a more aggressive phenotype, rapid onset of metastasis, shorter response duration to therapies, and worse prognosis (Yin et al., 2020).

The complex and heterogeneous nature of breast cancer and the contribution of Nrf2 are currently under investigation, and limited data are available to justify the major role of Nrf2 in breast cancer progression (Oshi et al., 2020). Several studies have proposed a link between the enhanced activity of Nrf2 and the potentiation of breast cancer metastasis. Nrf2 is present in the cytoplasm, where it binds to Kelch-like ECH-associated protein 1 to form a complex with Cul3 and Rbx1, which degrades the Nrf2 proteasomal enzyme (Bellezza et al., 2018). However, the stable Nrf2 heterodimerizes with Maf proteins in nuclei, enhancing the antioxidant property to protect the target gene (Pandey et al., 2017). Nrf2 is also associated with Notch pathways, which enhance the survival, invasion, and chemoresistance associated with tumor cells with abnormal expression of Nrf2 (Lamy et al., 2017). Moreover, Nrf2 has a key role in the activation of HIF1α, which is followed by enhanced glycolysis, which enhances breast cancer progression (Zhang et al., 2018).

Redox mechanism-based therapy is known to play an important role in cancer treatment; however, its utility is compromised with the inherent tendency to develop resistance over time. Here, Nrf2 is responsible for the regulation of antioxidant and cytoprotective properties through the activation of several genes involved in glutathione (GSH) synthesis and chemoresistance (Raghunath et al., 2018). The recent literature is flooded with multiple outputs by various researchers on the role of Nrf2 and its exact involvement in biological functions. In this article, we discuss the role of Nrf2 in the specific context of breast cancer, its development, angiogenesis, chemoresistance, stem cell generation, and metastasis. The effective treatment strategies are also elaborated and explained here for the treatment of breast cancer with abnormal Nrf2 expression.

## 2 Nuclear Factor Erythroid 2-Related Factor 2: General Mechanism/Pathways

Nrf2 is an omnipresent transcription factor that is essential for maintaining cellular homeostasis. It promotes the activity of cytoprotective genes such as glutamate cysteine ligase (GCS) and NAD(P)H:quinone oxidoreductase-1 (NQO1). Generally, Nrf2 is a transcription factor of the Cap n Collar (CNC) family and contains a basic leucine zipper region (bZip). Nrf2 is made up of 650 amino acid residues and has a molecular weight of 96–118 kDa because of posttranslational changes such as phosphorylation (Moi et al., 1994; Pi et al., 2007). Nrf2 tends to promote the transcription of genes by heterodimerizing with Maf proteins or other homologs to a cis-acting DNA transcriptional regulator, specifically the antioxidant response element (ARE) (Zhu et al., 2016). Nrf2 contains seven Neh domains (Neh1-Neh7) that are considered crucial for its action and suppression (Itoh et al., 1999). Neh2 and Neh6 are degron sections that are targeted by Keap1 and TrCP *via* 29DLG31/79ETGE82 motifs and 343DSGIS347/382DSAPGS387 motifs, respectively (McMahon et al., 2004; Chowdhry et al., 2013). Ablation or diminution with time is associated with an increase in oxidative stress and cellular death (Lee et al., 2003; Suh et al., 2004; Silva-Islas and Maldonado, 2018).

**FIGURE 1:**
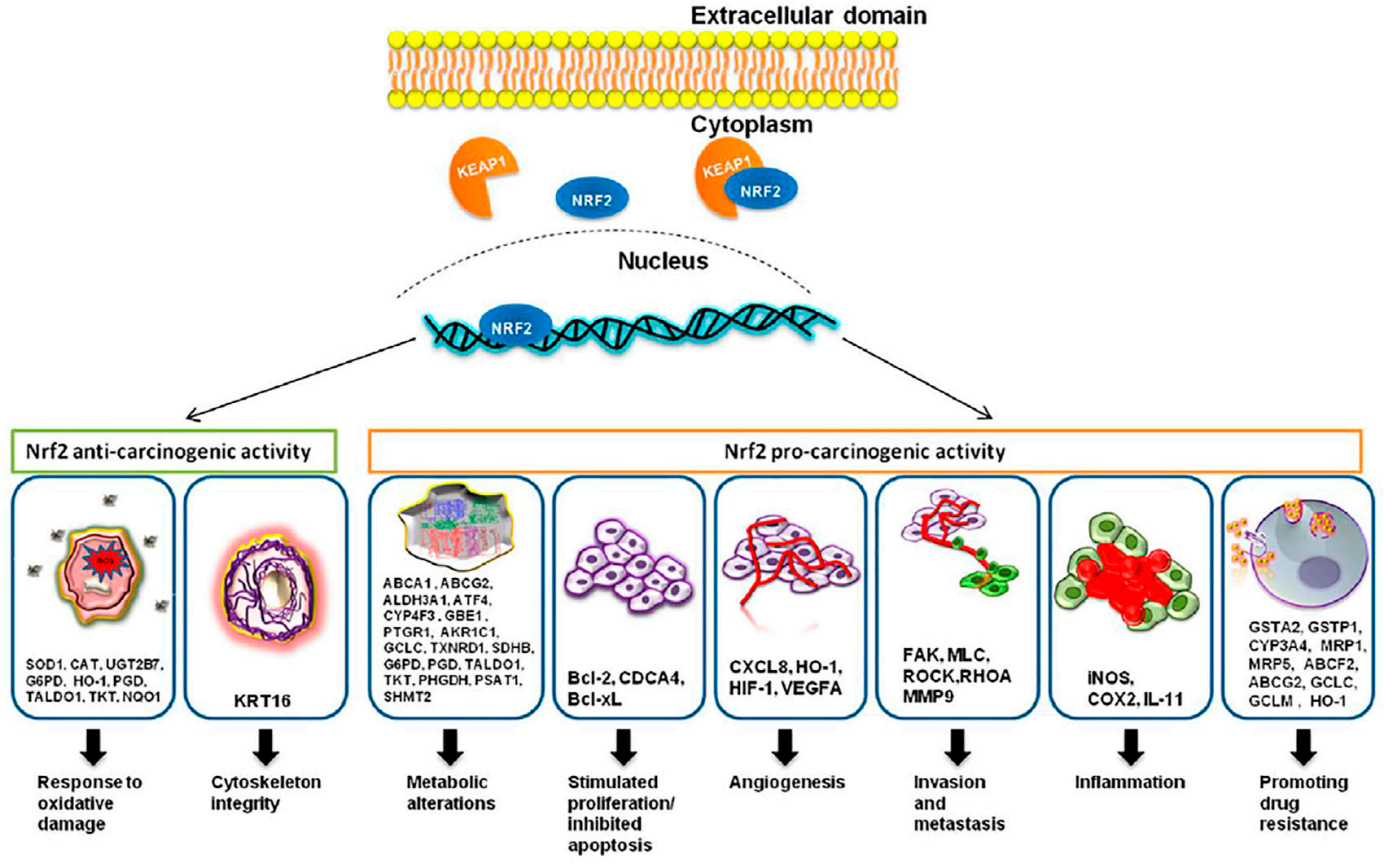
Nrf2, through its targeted genes, has an anti-carcinogenic role in the case of normal cells and a pro-carcinogenic effect in the case of transformed malignant cells. The image was acquired from (Zimta et al., 2019). Under creativecommons.org/licenses/by/4.0/.

Nrf2 is considered the key regulator of the oxidative cellular state through the interaction of the proteins CHD6, CBP, and RAC3; Neh3, Neh4, and Neh5 are transactivation domains (Katoh et al., 2001; Nioi et al., 2005; Kim et al., 2013) Finally, the Neh7 domain is linked to the RXRα protein for Nrf2 suppression (Wang et al., 2013). Nrf2 is expressed everywhere (Moi et al., 1994) and regulates the expression of approximately 1,055 genes (Malhotra et al., 2010) that contain the cis-acting antioxidant response element (ARE, 5′-GTGACNNNGC-3′) (Rushmore et al., 1991). They ARE sequence is located in the regulatory regions of genes involved in cellular growth, oxidation and detoxifying response, metabolic, immunologic response, cell survival, signaling, and cellular cycle (Figure 1).

Chemopreventive drugs activate Nrf2 (Fahey et al., 2002; Iida et al., 2004; Sussan et al., 2009) and pharmacological stimulation of Nrf2 has been extensively supported as a primary method for cancer and other illness prevention (Kwak et al., 2004; Zhang et al., 2004). Moreover, recent research reveals that Nrf2 activity may be elevated in cancer cells, and its cytoprotective action may promote cancer cell survival and proliferation, implying that inhibition of Nrf2 during cancer treatment may be essential (Lau et al., 2008; Kang et al., 2020). The mechanism by which chemopreventive drugs activate Nrf2 is, however, poorly understood. While most research has shown that chemopreventive drugs activate Nrf2 by preventing its protein degradation, there is also some research implying that Nrf2 gene transcription may be promoted (Kwak et al., 2002; Pi et al., 2003).

Keap1 reactive cysteine residues have a negative impact on Keap1-mediated Nrf2 enzymatic activity, which results in Nrf2 accumulation/activation and cytoprotection by enhanced ARE transcriptional genes (Dinkova-Kostova et al., 2005a). Furthermore, chemical alteration of Keap1 cysteines has been found to cause its own ubiquitination and destruction, sparing Nrf2 from destruction (Hong et al., 2005). Nonetheless, some studies suggest that chemically changing Keap1 cysteines are inadequate to interrupt the Nrf2 interaction with Keap1 (D. D. et al., 2004; Eggler et al., 2005), while others state that phosphorylation of Nrf2 (at Ser40) by protein kinase C or extracellular protein kinase PERK increases Keap1 dissociation (Huang et al., 2002).

Nrf2 has a key role in redox homeostasis through NADPH and ROS regeneration and glutathione (GSH) and thioredoxin (TXN) antioxidant synthesis. The regulation and maintenance of GSH synthesis is controlled by Nrf2 and by the expression of two types of subunits, the catalytic subunit (Gclc) and the modifier subunit (Gclm), which help in the synthesis of glutamate-cysteine ligase (Gcl) (Moinova and Mulcahy, 1999; Dinkova-Kostova and Abramov, 2015). Nrf2 regulates several GSHs (Gsta1/2/3/5, Gstam1/2/3, and Gstp1) and other ROS detoxifying enzymes (Thimmulappa et al., 2002). The TXN-based antioxidation system is also controlled and regulated by Nrf2. Nrf2 controls thioredoxin reductase 1 (Txnrd1) and sulfaredoxin, which are essential for the oxidized protein thiol reduction mechanism. The NADPH enzyme is an important factor for cytoprotection. Nrf2 positively regulates NADPH-generating enzymes, such as 6-phosphogluconate dehydrogenase (Pgd), isocitrate dehydrogenase 1 (Idh1), glucose-6-phosphate dehydrogenase (G6PD), and malic enzyme 1 (Me1) (Tonelli et al., 2018). Moreover, Nrf2 is also associated with the regulation of other cytoprotective enzymes. Hmox1 is a part of the heme oxygenase enzyme, which is involved in ferritin production by oxidizing free Fe^2+^ into Fe^3+^. Nrf2 regulates the expression of genes that are especially encoded ferritin complex constitutions (Wu et al., 2011). Nrf2 plays a major role in the cellular defense system by controlling xenobiotic and oxidative stress conditions by controlling the expression of antioxidants and detoxifying genes in normal cells (Tonelli et al., 2018).

The cellular Nrf2 level is quite low in normal unstressed situations, but it substantially increases when exposed to electrophilic compounds or reactive oxygen species (ROS) (Itoh et al., 1997). In Keap1, electrophiles alter reactive cysteine residues (Dinkova-Kostova et al., 2005b). Murine Keap1 has 25 cysteine residues, which are classified into different classes based on their reactivity to different electrophiles (Zhang and Hannink, 2003).

**FIGURE 2:**
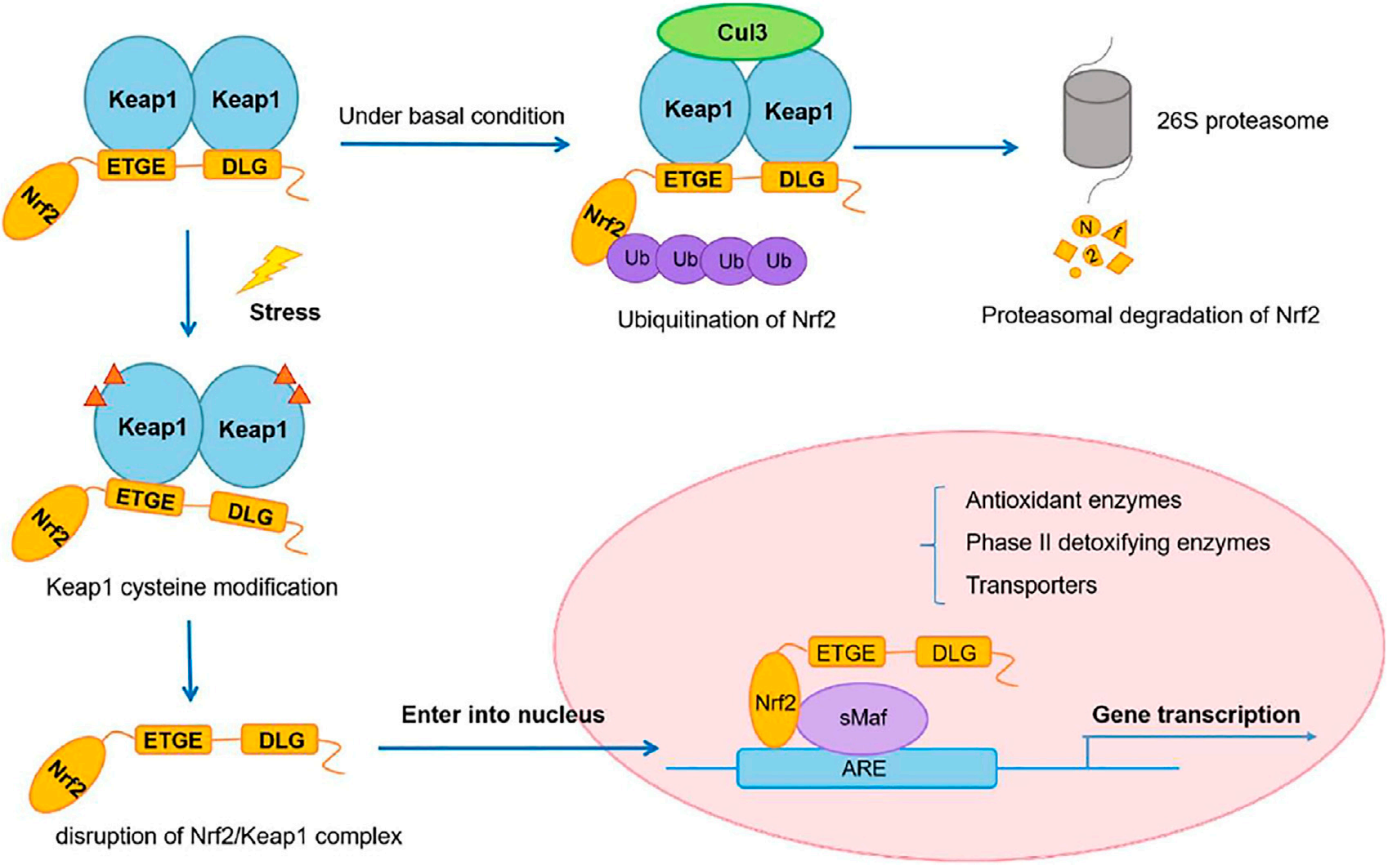
Nrf2/Keap1 signaling pathway. Adapted from (Wu et al., 2019). Under © 2019 The Authors. Cancer Medicine published by John Wiley & Sons Ltd. https://creativecommons.org/licenses/by/4.0/.

Cysteine 151 (C151) and C288, for example, have been proven to sense definite sets of electrophiles generated endogenously or exogenously (Eggler et al., 2008). Although particular cysteine residues changed by ROS have yet to be identified, oxidative alteration of Keap1 has been reported to reduce its binding to Nrf2 or CUL3. These electrophilic and oxidative changes inactivate Keap1, allowing Nrf2 to be stabilized. As a result, the rise in Nrf2 in response to electrophiles and ROS is not a precise induction but rather a process known as depression (from rapid degradation-based repression) (Taguchi and Yamamoto, 2017) (Figure 2).

## 3 Nuclear Factor Erythroid 2-Related Factor 2 in Breast Cancer

Nuclear factor erythroid 2-related factor 2 (Nrf2) is primarily responsible for the cytoprotection of normal cells by detoxifying mechanisms through oxidation, electrophilic stress, or xenobiotic processes. The abnormal expression of Nrf2 in cancer cells leads to a pro-oncogenic program that stimulates the malignancy of cancerous cells/tissue. An increased level of Nrf2 expression resulted in lower survival and increased cancerous cell progression and proliferation in breast cancer patients (Almeida et al., 2020). Convening evidence from recent decades indicates that Nrf2 can exert chemopreventive properties on normal cells through an ROS-dependent oxidation process. However, aberrant expression in breast cancer imparts cytoprotective effects to cancerous cells by suppressing ROS-dependent DNA damage and carcinogenicity.

Nrf2 has dual roles as a pro-oncogenic and anti-oncogenic in breast cancer cells and healthy cells, respectively. The dual role contributed by the transcription factors depends on metabolic adaptation, cell proliferation, and induction of Nrf2 (Lee et al., 2018; Aliyev et al., 2021). For example, De Blasio and his co-workers demonstrated that Nrf2 upgraded both the proliferation and antioxidant capacity in triple-negative breast cancer (TNBC) cells by downmodulating miR-29b-1-5p expression. miR-29b-1-5p is a prognostic biomarker in basal-like breast cancer that produces cytotoxic events through decreased levels of p-AKT and p-Nrf2 and inhibition of N-methyltransferase expression. Thus, it helps to reduce cell proliferation and invasion. They showed in their research that the activation of miR-29b-1-5p expression inhibits the expression of AKT, which could suppress Nrf2 (De Blasio et al., 2020). Nrf2 could be one of the major hallmarks in the development and regulation of breast cancer. It was also suggested that Nrf2 is highly expressed in ER-negative breast cancer. Nrf2 downregulates CXCL13, which suppresses breast cancer proliferation. The increased level of CXCL13/CXCR5 coarticulation in ER (+) breast cancer cells with lower Nrf2 levels helps advance tumor intrusion and metastasis (Aliyev et al., 2021).

The involvement of Nrf2 and its role in different subtypes in breast cancer are still under investigation. To date, no data have been published explaining the level of Nrf2 expression in different subtypes of breast cancer. However, few published studies have shown that the Nrf2 and keap-1 pathways are more highly activated in breast cancer, which has ER, PR, and HER receptor positivity, than in TNBC (Karihtala et al., 2011). The increased level of Nrf2 causes lower overall survival and disease-free survival in all breast cancer patients. However, normal cells can exert chemopreventive effects *via* Nrf2. Due to the dual role of Nrf2 (pro-oncogenic and anti-oncogenic) in cancer patients, other factors, such as metabolic genes, proliferative genes, and angiogenesis genes, should also be considered for inhibiting Nrf2 through Nrf2 inhibitors (Almeida et al., 2020; Smolková et al., 2020; Aliyev et al., 2021).

## 4 Role of Nuclear Factor Erythroid 2-Related Factor 2 in Breast Cancer Resistance

**FIGURE 3:**
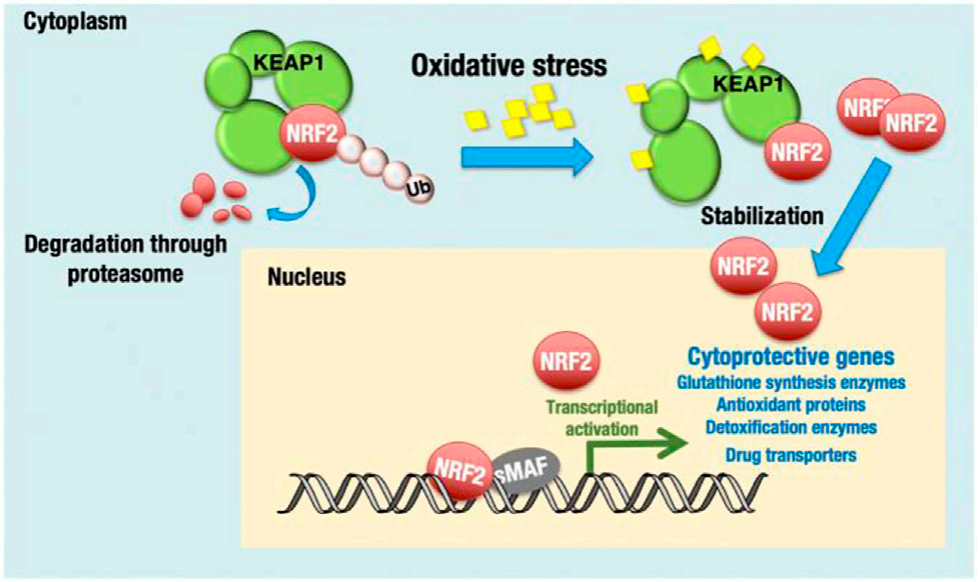
KEAP1-NRF2 system for oxidative stress response. NRF2 is a transcription activator and regulates many cytoprotective genes. Under unstressed conditions, Nrf2 is bound by KEAP1 and ubiquitinated for degradation. Adapted from (Okazaki et al., 2020). Under © 2019 The Authors. https://creativecommons.org/licenses/by/4.0/.

Nrf2 is a leucine zipper protein transcription factor that positively regulates the expression of antioxidant genes, such as GPX4, HO-1, SLC7A11, and NAD(P)H quinone oxidoreductase (Qiao et al., 2020). As discussed above, Nrf2 regulates oxidative stress and protects cancer cells by the toxic effect of therapeutic drugs/anticancer drugs. Nrf2 is a central transcriptional activator with an active role in cellular defense mechanisms against electrophilic or oxidative stress (Uruno and Motohashi, 2011) (Figure 3). Studies have reported that Keap1 constantly degrades Nrf2, which downregulates Nrf2 by the ubiquitin–proteasome pathway under normal cellular conditions. At the somatic mutation stage, Keap1 is inactivated, which enhances Nrf2 expression in the nucleus and induces cytoprotective genes such as heme oxygenase-1 [HO-1 (HMOX1)] and NAD(P)H:quinone oxidoreductase 1 (NQO1) (Baird and Dinkova-Kostova, 2011). Nrf2 overexpression in the protein–protein interaction region compromised the Keap1 checkpoint, resulting in epigenetic and posttranscriptional modification and enhancing proto-oncogenes (Sporn and Liby, 2012). Moreover, Nrf2 phosphorylation and polymorphisms in Nrf2 cause poor prognosis in breast cancer (Hartikainen et al., 2012; Ishikawa, 2014).

Nrf2 overexpression in breast cancer also improves the excess expression of P53 in an inhibitor protein for stimulating apoptosis, also known as Rel-A inhibitor, which promotes cancer development and tumor-associated drug resistance. The higher P53 level in breast cancer cells restricts the binding of free Keap1 to free Nrf2; therefore, the level of free Nrf2 is higher in the nucleus and induces chemoresistance (Ge et al., 2017). Few more studies have suggested that other proteins, such as p21 or p62, also restrict the Keap1–Nrf2 interaction and induce Nrf2-associated drug resistance in tumor cells (Chen et al., 2009; Lau et al., 2010).

## 5 Role of Nuclear Factor Erythroid 2-Related Factor 2 in Metastasis

In response to various oxidative-driven transcriptional processes, Nrf2 combines physiological stress signals by interacting with antioxidant response domains within the regulatory regions of Nrf2-controlled genes (Itoh et al., 1999; Jaiswal, 2004; Nguyen et al., 2009). Because of its cytoprotective role, Nrf2 has been identified as a tumor suppressor, and its activity can also prevent tumor growth. For example, sulforaphane, a Nrf2 inducer, has also been found to prevent the development and metastasis of effectively supported implanted breast cancer cells in female athymic mice as well as to reduce the growth of human breast cancer cells (Kanematsu et al., 2011). Moreover, several researchers have demonstrated abnormally active Nrf2 in several breast cancer cells (Nioi and Nguyen, 2007; Syed Alwi et al., 2012; Zhong et al., 2013), and recent genetic investigations of breast cancers revealed the crucial role of Nrf2 in oncogenesis (Hayes and McMahon, 2009; Denicola et al., 2011).

In breast cancer, Nrf2 activation enhances Rho expression and downstream proteins such as focal adhesion kinase 1 (FAK), modulator of volume-regulated anion channel current 1 (MLC), and Rho-associated coiled-coil-containing protein kinase 1 (ROCK), whereas it lowers estrogen-related receptor (ERR1) expression. Nrf2 has a direct interaction with the BRCA1 susceptibility protein, resulting in enhanced BRCA protein stability. Estrogen improves Nrf2 activation in the absence of BRCA expression, leading to reduced ROS production and enhanced cytoprotection (Gorrini et al., 2013). Exogenous antioxidants such as phospholipid hydroperoxide glutathione peroxidase (PHGPx) or pro-oxidant 15-lipoxygenase (15-LOX) decrease the levels of vascular cell adhesion molecule (VCAM) through an interaction with Nrf2 in the gene promoter of this locus (Zimta et al., 2019).

RhoA is a member of the Ras superfamily, which regulates cell migration and invasion of cancer cells (Pertz et al., 2006; Vega and Ridley, 2008). RhoA GTPases regulate the formation of actin stress fibers and limit the size of the lamellipodium through their downstream effectors mDIA and ROCKs, which cycle between an inactive GDP-bound and an active GTP-bound form (Riento and Ridley, 2003; Worthylake and Burridge, 2003). The RhoA regulatory effect is controlled to the extent of protein stability and deterioration (Nethe and Hordijk, 2010). However, no constitutively active Rho GTPase mutations have been found in human cancers (Rihet et al., 2001; Fritz et al., 2002), and clinical and experimental studies show a link between enhanced RhoA expression and poor clinical outcome in breast cancer (Bellizzi et al., 2008; Chan et al., 2010; Ma et al., 2010). High levels of Nrf2 are associated with tumorigenesis and poor prognosis, and it promotes RhoA expression by interacting with and silencing the ERR1 gene, allowing breast cancer cells to proliferate and metastasize. In conjunction with published reports, deactivating Nrf2 could be advantageous in breast cancer treatment in the clinical stage (Zhang C. et al., 2016).

## 6 Role of Nuclear Factor Erythroid 2-Related Factor 2 in Tumor Angiogenesis

Due to excessive ROS generation, by interacting with the Notch/delta-like 4 (Dll4) system, Nrf2 promotes vascular sprouting by reducing the impairment of vascular signal transduction and angiogenesis and regulating the production of tip cells (Wei et al., 2013). Its role in microvascular epithelial cell migration and blood vessel generation has been verified, which suggests the development of a VEGF-Nrf2 positive loop (Li et al., 2016). The positive regulation of endothelin receptor B by nitro-oleic acid (OA-NO2) was substantially regulated by Nrf2, suggesting that Nrf2 silencing enhanced endothelin-1 levels in the circulation (Kansanen et al., 2017). ROS have been demonstrated to be proangiogenic mediators at adequate levels through a different pathway, including increasing VEGF and angiopoietin-1/Tie-2 signaling (Harel et al., 2017; Zou et al., 2019). Under oxidative stress, Nrf2 plays a direct or indirect role in angiogenesis control (Guo and Mo, 2020).

Disturbances in proliferation and apoptosis have an important role in tumor and angiogenesis. Past studies indicate that Nrf2 is required for angiogenesis of normal vasculature. Valcarcel-Ares et al. published that short interfering RNA (siRNA) and Keap1 were involved in silencing the expression of Nrf2 in coronary arterial endothelial cells, leading to cellular proliferative capacity impairment, improper adhesion to extracellular matrix proteins, reduced migration, and impaired capillary formation (Valcarcel-Ares et al., 2012). It is believed that the Nrf2 in breast tumor angiogenesis affects the biological behavior of intratumoral endothelial cells (Zhou et al., 2012).

Nrf2 regulates redox homeostasis and is associated with cellular growth and malignancy (Denicola et al., 2011; Mitsuishi et al., 2012). Neovascularization is essential for organ regeneration, tissue repair, and embryogenesis (Hoeben et al., 2004). Tumor growth is linked to angiogenesis, and in malignant tumors, the exchange of oxygen/carbon dioxide and nutrients/waste products depends on blood vessels. Angiogenesis plays a critical role in the migration and invasion of primary malignancies to distant areas of the body (Carmeliet, 2005). Oxidative stress affects angiogenesis in atherosclerosis, ocular diseases, and tumorigenesis (Chung and Ferrara, 2011; Coso et al., 2012).

Another important factor in angiogenesis is hypoxia, as it activates angiogenesis mediators such as HIF and VEGF transcription factors, which are interlinked to tumor dissemination, invasion, and metastasis (Lee et al., 2009). In addition, elevated levels of peroxides also trigger tumor angiogenesis (Szatrowski and Nathan, 1991)**.** Various studies have shown that Nrf2 participates in angiogenesis regulation. Hypoxia triggers the Nrf2/ARE pathway, which promotes tumor blood vessel development. The forceful blocking of HIF-1 signaling in the absence of Nrf2 can result in a decrease in capillary density (Zhang et al., 2013). Kweider et al. (2011) explored the role of VEGF in cancer cell proliferation. Another study found a link between VEGF and Nrf2 activation, demonstrating that VEGF increased Nrf2 expression in an ERK1/2-dependent manner (Kweider et al., 2011). Shao et al. reported that curcumin upregulates Nrf2 and GSH and causes ROS scavenging, reduces the expression of VEGF, and inhibits hepatocarcinoma angiogenesis and invasion (Shao et al., 2019). As a result, VEGF and Nrf2/HIF-1 facilitate tumor angiogenesis.

The activation of HIF-1α through Nrf2 also enhances the angiogenesis and progression of breast cancer. HIF-1α is the major transcription factor responsible for adaptive hypoxic conditions and regulates metabolic genes (such as GLUT1, HK2, and PGK1), angiogenesis genes (such as VEGF and FGF), and apoptosis genes (e.g., Bax, BCL2, and P53). The inhibition of the aberrant expression of Nrf2 could be effective in breast cancer treatment. This experiment was performed by Zhang and his coworkers in 2018, who found higher levels of Nrf2 and HIF-1α mRNA and proteins in MCF-7 and MDA-MB-231 breast cancer cells than in normal breast cells (MCF-10A). The knockdown of Nrf2 overexpression decreased HIF-1α mRNA levels and reduced breast cancer cell proliferation (Zhang et al., 2018).

## 7 Role of Nuclear Factor Erythroid 2-Related Factor 2 in Breast Cancer Stem Cells

The studies found and identified Nrf2 as a major regulator of chemoresistance in cancer stem cell (CSC)-enriched breast cancers (Achuthan et al., 2011; Ryoo et al., 2015) as well as the activation of Nrf2-associated antioxidant genes such as HO-1, NQO1, Prx1, and others that leads to radioresistance in several other cancer cells (Zhou et al., 2013). Because breast cancer stem cells (BCSCs) have low ROS levels and increased antioxidant defense (Lobo et al., 2009), the involvement of the Nrf2 pathway in BCSC radioresistance requires further investigation.

For example, an enhancement in ALDH levels in BCSCs causes higher radioresistance, carcinogenesis, decreased apoptosis, and regulation of signaling pathways that enhance mesenchymal–epithelial transition and migration. Moreover, following fractionated irradiation, tumorigenicity was increased. Further examination of the involvement of Nrf2 in radioresistance revealed that following irradiation, Nrf2 and its related genes HO-1 and NQO1 were significantly elevated. All of the foregoing pathways of radioresistance in BCSCs were reduced by shRNA-mediated knockdown of Nrf2 expression. The process of Nrf2 activation was reported to be regulated by Keap1 silencing, as there was no change in GSK-3 or Bach1, a negative regulator of Nrf2. We also found no change in the methylation status of the Keap1 promoter; however, we identified a substantial rise in the expression of miR200a. This suggests that miR200a might be a mechanism for Keap1 silencing. This work offers data for the significance of Nrf2 and its downstream genes in radioresistance in BCSCs and identifies processes by which the Nrf2/Keap1 pathway influences radioresistance in BCSCs (Kamble et al., 2021).

Evidence gathered to date suggests that the upregulation of Nrf2 promotes tumor growth and survival by creating a favorable environment for cancer stem cells. The direct involvement of Nrf2 in cellular ROS regulation and anticancer drug resistance is a potential contribution of Nrf2 to CSC biology. Wu et al. (2015) recently demonstrated that Nrf2 activation was associated with CSC-enriched spheroid breast cells (Wu et al., 2015). Another published report demonstrated that Nrf2 activation is characterized in a CD44-overexpressing breast CSC-like system and investigated the direct link of Nrf2 with the CSC phenotype (Ryoo et al., 2018).

Similarly, in another work, it was suggested that intracellular ROS generation is low and is upregulated by Nrf2-induced GCLC expression, which allows the self-renewal of CSCs through the Fork head box O3a-Bmil-axis (Kim et al., 2020). Furthermore, it has also been demonstrated that hypoxia-driven CSC enrichment in breast cancer originates from a dedifferentiation process and that hypoxia-inducible factors (HIFs) are essential for chemotherapy resistance in breast CSCs (Iriondo et al., 2015). Surprisingly, differentiating CSCs exhibit multidrug resistance (MDR) because of the PERK-Nrf2 signaling pathway (Del Vecchio et al., 2014).

## 8 Strategies to Overcome Breast Cancer Resistance

### 8.1 Endogenous Molecule Inhibitors

Various endogenous molecules, such as E-cadherin (Kim WD. et al., 2012)**,** activating transcription factor-3 (ATF3) (Brown et al., 2008), tumor protein P53 (Faraonio et al., 2006), BTB and CNC homology 1 (bach1) (Dhakshinamoorthy et al., 2005), caveolin-1 (Li et al., 2012), and GSK3 (Chowdhry et al., 2013), play a role in the downregulation of Nrf2. These molecules disrupt the Nrf2/ARE pathway and its expression levels. Specifically, E-cadherin has a role in chemoresistance and enables Keap1 to reduce endogenous Nrf2 levels by recruiting Nrf2 *via* β-catenin. P53 activation causes downregulation of Nrf2 and generates higher levels of ROS, which promotes apoptosis (Faraonio et al., 2006). These molecules are generally required for normal cell homeostasis; therefore, disruption of the signaling pathway would be based on a thorough understanding of cellular biology and events before beginning this therapy.

### 8.2 Exogenous Natural Inhibitors

The complex tumor microenvironment limits the application of endogenous inhibitors; hence, exogenous inhibitors are emerging molecules. Some exogenous natural inhibitors not only inhibit Nrf2 but also promote anticancer drug sensitivity in resistant cancerous cells. For example, vitamin C could decrease oxidative stress in the tumor microenvironment, which suppresses the translocation of Nrf2 to the nucleus from the cytoplasm. Ascorbic acid (vitamin C) is a reducing agent that binds to ARE, inhibits Nrf2 translocation to DNA, and decreases the levels of g-GCSl mRNA and GSH. Furthermore, vitamin C in combination helped in the reversal of drug resistance associated with imatinib treatment (Tarumoto et al., 2004). Similarly, Xiu et al. (2007) reported that all-trans retinoic acid (ATRA) prevents binding of Nrf2 to ARE by forming a complex with RARa (Xiu et al., 2007). In another example, trigonelline, a coffee-derived alkaloid, also lowers Nrf2 levels in drug-resistant pancreatic cancer cell lines by blocking Nrf2-dependent proteasomal gene expression of s5a/psmd4 and a5/psma5 and reducing proteasome activities. Trigonelline also reduced basal and tert-butylhydroquinone-induced Nrf2 activity and reduced the drug resistance induced by higher levels of Nrf2 (Arlt et al., 2013).

Nrf2-resistant cancer cells were observed to have higher levels of Nrf2 and other associated genes, such as NQO1, MRP-1, HO-1, CGLM, and CGLC. The expression of these genes is highly linked with the development of drug resistance in lung cancer. Cryptotanshinone treatment was administered in combination with cisplatin to the cells, and it was found that Nrf2 and its associated target gene expression were diminished in cisplatin-resistant lung cancer. Furthermore, it was also observed that cryptotanshinone affects other signaling pathways, such as the MAPK, Akt, and Stat3 (Xia et al., 2015). Luteolin, a vegetable-derived flavonoid, dramatically induces Nrf2 mRNA degradation and other downstream ARE-driven genes, such as NQO1, HO-1, and AKR1C (Tang et al., 2011). As a result, luteolin induced cell death when used in combination with oxaliplatin, bleomycin, and DOX. In TNBC cells, luteolin-loaded nanoparticles reduced the Nrf2, HO1, and MDR1 mRNA expression levels. In addition, luteolin nanoparticles improved doxorubicin sensitivity in MDA-MB-231 cells (Sabzichi et al., 2014).

A few other flavonoids, such as chrysin (Gao et al., 2013a), apigenin (Gao et al., 2013b), wogonin, and 30, 40, 50, 5,7-pentamethoxyflavone (PMF) (Hou et al., 2015), can also inhibit Nrf2 expression in cancerous cells and produce apoptotic effects. Flavonoids are known for their antioxidant and cytoprotective properties, and their Nrf2-inducible effect has been observed in a few studies (Bai et al., 2016). Nanocarriers entrapping flavonoids were developed for targeted drug administration, enhancing the bioavailability of poorly water-soluble medications and delivering macromolecules to the cell’s site of action. Moreover, by combining therapeutic agents with imaging tools that can visualize the drug delivery location and coadministration of two or more medicines, ADRs can be reduced, and nanotechnology plays a key role in this (Kumar et al., 2016; Khan et al., 2021).

### 8.3 Inhibitors of Nuclear Factor Erythroid 2-Related Factor 2 in Cancer Therapy

Nrf2 regulates genes such as transporters, phase II detoxifying enzymes, and endogenous antioxidants by controlling cellular defense response mechanisms. The literature shows the role of Nrf2 in chemoresistance, and its expression has been identified in many types of cancer (Samadi et al., 2014). High-throughput screening (HTS) in combination with cell-based assays has proven to be a potential approach to discover new anticancer drugs and to identify therapeutic uses of compounds that are approved by the FDA. Plant extracts and other phytochemicals have anticancer activity and are under treatment regimens or in clinical trial investigations.

Procyanidin CCE lowers the levels of Nrf2 expression and inhibits cell growth in the case of cancer. Another compound based on a flavonoid, luteolin, present in fruits and vegetables, inhibits Nrf2 in cancer cells (Choudhari et al., 2020). Similarly, trigonelline, an alkaloid present in hemp seed, coffee beans, oats, garden peas, and fenugreek seed, shows high basal Nrf2 activity that protects against etoposide- or TRAIL-induced apoptosis by elevating proteasomal gene expression (Panieri and Saso, 2019). Brusatol, a quassinoid *Brucea javanica* plant extract, shows antitumor activity (Yu et al., 2020). Chrysin, a bioflavonoid, protects against carcinogenesis by decreasing the mRNA and protein levels of Nrf2 (Wang et al., 2018). Apigenin, a dietary flavonoid present in fruits and vegetables, is said to exhibit anticancer effects *in vitro* and *in vivo* (Yan et al., 2017). Oridonin, a diterpenoid derivative, possesses anticancer effects in solid and hematologic tumors (Lu et al., 2018). Similarly, Honokiol, a lignan isolated from *Magnolia*, produces toxicity in lymphoid cancer cell lines. Honokiol lowers NF-κB activity and Nrf2 proteins, resulting in higher ROS production and apoptosis (Ong et al., 2019). Halofuginone inhibits and activates Nrf2 constitutively in resistant cancer cells (Panieri and Saso, 2019). Another anticancer agent, plumbagin, a naphthoquinone, induces oxidative stress-dependent Nrf2 activity in cancer cells (Kapur et al., 2018). Berberine is an alkaloid found in various medicinal plants. By inducing oxidative stress, berberine has anticancer properties in breast cancer (Lu et al., 2012). Parthenolide, a sesquiterpene lactone found in medicinal plants, shows anticancer effects by modulating ROS (Mathema et al., 2012). Wogonin, a flavonoid obtained from *Scutellaria baicalensis* Georgi, also reduced Nrf2 nuclear content (Zhong et al., 2013).

The extract of chestnut leaf works by suppressing the Nrf2-mediated antioxidant system and may increase ROS production and thereby promote paclitaxel-induced apoptotic cell death. The results showed that treatment with chestnut leaf extract reduced the expression ratio of Bcl-2 and Bax, and an increase in the amount of cleaved PARP and paclitaxel-treated CSCs resulted in significant mitochondrial damage compared to untreated or extract-treated CSCs. This result suggests that the combination of paclitaxel with chestnut leaf extract can effectively eliminate paclitaxel-induced CSCs. As a result, when chestnut leaf extract and paclitaxel are in combination, synergistic effects are produced; however, to determine possible adverse effects, such as counteraction or additive toxicity of the drug because of chestnut leaf extract and paclitaxel, further studies need to be performed (Scarpa and Ninfali, 2015; Woo et al., 2017).

The therapeutic role of various polyphenols in breast cancer has also been established. Curcumin activates Nrf2, which promotes the expression of antioxidative enzymes such as NQO1, HO-1, GST, and glutathione reductase (GR) and induces cellular senescence (Das and Vinayak, 2015). Curcumin’s activation of Nrf2 relies on the thiol modulation of KEAP1 (Shin et al., 2020). Similar to curcumin, other polyphenols induce Nrf2 and downstream genes, primarily phase II detoxification enzymes (Foygel et al., 2015). In MCF-7 and MDA-MB-231 breast cancer cells, NRF2 induction by EGCG was examined using Western blot analysis (Hu et al., 2010). The biphasic effects of the grapefruit polyphenol resveratrol are well known. Resveratrol therapy increases cell growth in breast cancer cells at low doses, while it causes cytotoxicity at higher concentrations. Similarly, resveratrol exhibits antioxidant properties at low concentrations while exhibiting a prooxidant profile at larger doses. Rai et al. used resveratrol in the 50–400 M concentration range to treat MCF-7 and MDA-MB-231 cells, which displayed high cytotoxicity in a dose-dependent manner (Rai et al., 2016).

The therapeutic role of melatonin in breast cancer is also known. Melatonin is an indole pineal hormone. Melatonin synthesis and secretion have been shown to be substantial contributing factors for breast cancer growth and progression (Jasser et al., 2006; Hill et al., 2015). Melatonin’s involvement in the protection and treatment of breast cancer, particularly the control of oxidative stress, has been widely researched (Gurer-Orhan et al., 2018), and the regulation of miRNAs is linked to apoptosis, cellular senescence, and proliferative genes (Chuffa et al., 2020). According to oxidative stress–mediated physiological responses, melatonin activates Nrf2 by upregulating cellular mediators such as PKC (Li et al., 2018), SIRT1 (Shi et al., 2019), and PI3K/AKT (Zhang et al., 2017).

Specific to breast cancer, metformin has been shown to target miRNAs, proteins involved in miRNA biogenesis, and target genes in CSCs. Metformin suppresses breast cancer cell proliferation by downregulating miR-27a (Zhao et al., 2016) and upregulating miR-193 (miR-193a-3p and miR-193b), which increases AMPK and decreases FASN levels, respectively (Wahdan-Alaswad et al., 2014). It also increases the expression of let-7a (a tumor suppressor miRNA) while decreasing TGF-induced miR-181a (an oncogenic miRNA) production in MCF7 cells (Oliveras-Ferraros et al., 2011). Metformin’s anticancer activities in renal and breast cancer cells have been linked to the overexpression of miR-34a, which lowers cell proliferation and the Sirt1/Pgc1/Nrf2 pathway, respectively (Do et al., 2014; Xie et al., 2017; Saini and Yang, 2018).

## 9 Roles of Nuclear Factor Erythroid 2-Related Factor 2 Inhibitors and Inducers in Breast Cancer

In order to enable the readers to understand the anti-oncogenic and pro-oncogenic mechanisms of Nrf2 with various compounds, the information from various references been collated in Tables 1–3. The information has been segregated under subheadings of *in vitro/in vivo*/clinical studies.

**TABLE 1:** Anti-oncogenic and pro-oncogenic mechanisms of Nrf2 with various compounds: *in vitro* studies.

Mechanism	Effect	Compound	Reference(s)
**Anti-oncogenic role**			
Suppress Nrf2-regulated activity and Nrf2 expression in human A549 NSCLC cells	Promotes proteasome-independent Nrf2 degradation through IGFIR phosphorylation	Procyanidins from CCE	Ohnuma et al. (2015)
Blocks Nrf2 transcriptional activity and sensitizes Kap1-deficient cells to chemotherapeutics. ML385 interacts with the DNA-binding domain of NRF2 and most likely prevents the binding of Nrf2 to AREs	Impairs the DNA interaction of the MAGF–Nrf2 complex	ML385	Singh et al. (2016)
Inhibits Nrf2, increasing their sensitivity to several anticancer drugs	Decreases Nrf2 mRNA and protein levels	Luteolin	Chian et al. (2014)
Reduces the Nrf2 protein content in a KEAP1-independent way and decreases the expression of genes related to the MDR family	Promotes Nrf2 degradation	Brusatol	Olayanju et al. (2015)
Suppresses Nrf2 nuclear accumulation and the proteasome activity, abrogating their protective effects	Decreases the nuclear level of Nrf2	Trigonelline	Arlt et al. (2013)
Inhibits the proliferation, migration, and invasiveness by decreasing Nrf2 nuclear translocation and suppressing the expression of both *HO-1* and *NQO1*	Decreases Nrf2 mRNA and protein content, decreases Nrf2 nuclear translocation	Chrysin	Wang et al. (2018)
Antineoplastic activity in breast cancer by inducing oxidative stress	Promotes GSK -3β β-TrCP-dependent Nrf2 degradation	Berberine	Tang et al. (2009)
Promoting Nrf2 downregulation and increased ROS production, presumably by enhancing its ubiquitination and proteasomal degradation	Decreases Nrf2 expression	Parthenolide	Zunino et al. (2007), Mathema et al. (2012), Ghantous et al. (2013)
Prevented the Nrf2 nuclear translocation, promoting ROS-dependent cell death and increased susceptibility to common anticancer drugs, by also reducing the activity of MRPs	Decreases Nrf2 content at the transcriptional level, increases Keap1 levels	Wogonin	Sun et al. (2010), Zhong et al. (2013)
Reduced Nrf2 levels	Decreases Nrf2 mRNA and protein content	Apigenin	Gao et al. (2013b)
**Pro-oncogenic role**			
Inhibits proliferation and induces apoptosis in many kinds of cancerous cells	Pro-apoptotic effect has been hypothesized to mainly include inhibition of the NF-κB signaling pathway, inhibition of the cell cycle transit from G1 phase to G2 phase, inhibition of tumor angiogenesis by suppressing the phosphorylation of VEGFR-2, inhibition of P-glycoprotein	Wogonin	Huang et al. (2012)
Inhibition of proliferation and apoptosis	Suppression of pro-carcinogenic regulatory mechanisms and cell proliferation, modulation of intercell communication signals, destruction or removal of tumor cells, and induction of apoptosis	Luteolin	Seelinger et al. (2008)
Excellent inhibitory effect on both proliferation and metastasis of breast cancer	Sphere formation ability, proliferation, and migration are substantially suppressed, which can be attributed to the inhibitory effect of CHM-04 on EGFR	Chrysin	Moghadam et al. (2020)

**TABLE 2:** Anti-oncogenic and pro-oncogenic mechanisms of Nrf2 with various compounds: *in vivo* studies.

Mechanism	Effect	Compound	Reference(s)
**Anti-oncogenic role**			
Induction of phase-2 enzymes such as GST and UDP-glucoronosyl transferase	Triggered expression of Nrf2 increased the ARE binding affinity, which was consequently involved in the carcinogen detoxification and promoted oxidative stress	Sulforaphane	Kensler et al. (2000)
Induction of HO-1	Curcumin induced the HO-1 and its activity which alters the Nrf2–Keap-1 interaction which translocates Nrf-2 to the nucleus and initiates transcription of genes for detoxifying enzymes and cyto-protective proteins by ARE	Curcumin	Balogun et al. (2003), Pae et al. (2004)
Inhibition of benzo(a)pyrene-induced enzyme activity, cytochrome P450 1A1/2	Curcumin exhibits the anticarcinogenic effect by alteration of phase 1 and phase 2 regulating gene transcription, which enhances the binding of Nrf2 to ARE in the nucleus and promotes detoxifying activity	Curcumin	Garg et al. (2008)
↑GST, glutathione peroxide, HO-1	Enhanced the ROS-mediated autooxidation	Epigallocatechin-3-gallate	Wu et al. (2006)
Induction of phase-2 enzymes	6-HITC–dependent detoxification through ARE by enhanced Nrf2 localization at the nucleus	Wasabi	Morimitsu et al. (2002)
↑level of NQO1 and GST, UGT1A6 and GCLC mRNA expression	The upregulation of NQO1 induces oxidative stress and Nrf2-dependent transcription activation, which provides detoxification effect	Cafestol and kahweol	Cavin et al. (2002), Higgins et al. (2008)
↑Keap1-Nrf2 transcription by binding Keap1 cysteine residue, ↑level of GST	Carnosic acid induced the oxidative stress and excitotoxicity to provide cyto-protective effect in mice	Carnosic acid	Satoh et al. (2008)
↑IFN-gamma, ↑COX-2, ↑NQO1	Oleanolic acid suppresses the inducible nitric oxide synthase and blocks the inflammatory action by using the ARE-Keap1-Nrf2 signaling pathways	Oleanolic acid	Dinkova-Kostova et al. (2005c)
**Pro-oncogenic role**			
↓ARE binding affinity, ↑ERK expression	The increased ERK level suppresses the ARE activity and GCLC level which reduced the role of Nrf2 and ARE in cancer prevention	Tamoxifen	Kim et al. (2008)
↑Nrf2-DNA binding	The redox activation by ascorbic acid inhibited the Nrf-2–mediated gene expression	Vitamin C	Tarumoto et al. (2004)
↑ level of Prx1, GPx, and TrxR	The aberrant level of HO-1 promotes the Nrf2 downregulating genes, which contributed to the chemo preventive action and cancer promotion	HO-1 siRNA, Sulforaphane, tert-butylhydroquinone, and β-naphthoflavone	Li and Johnson, (2002), Hintze et al. (2003), Campbell et al. (2007), Rushworth and MacEwan, (2008)
↓GST, ↓GCLC, ↑NOQ1↑	In the case of aberrant expression, the oxidative stress inducible genes such as GST and GCLC cause the drug resistance to the alkylating agents	Alpha-tocopherol-hydroquinone and ubiquinol	Black and Wolf, (1991), Nioi and Hayes, (2004)

**TABLE 3:** Anti-oncogenic and pro-oncogenic mechanisms of Nrf2 with various compounds: clinical studies (Su et al., 2018; Goossens and Bailly, 2019; Robledinos-Antón et al., 2019; Nandini et al., 2020).

Compound	Mechanism of action	Effect	Disease	Clinical trial	Clinical trials identifier
Ursodiol (Goossens and Bailly, 2019)	Electrophilic modification of KEAP1 -Cys-151	It exhibits both pro- and anti-apoptotic properties toward different cell types. In particular, the UDCA drug can protect epithelial cells from damages and apoptosis while inducing inhibition of proliferation and apoptotic and/or autophagic death of cancer cells	Chronic hepatitis C	Phase 3	NCT00200343
			Primary biliary cirrhosis	Phase 4	NCT01510860
Oltipraz (Robledinos-Antón et al., 2019)	Electrophilic modification of KEAP1 -Cys-151	NRF2 inducer that enhances GSH biosynthesis and phase II detoxification enzymes, such as NQO1	Lung cancer	Phase 1	NCT00006457
			Nonalcoholic steatohepatitis	Phase 3	NCT0206339
Sulforaphane	Electrophilic modification of KEAP1 -Cys-151	Exerts its anticancer effects by modulating key signaling pathways and genes involved in the induction of apoptosis, cell cycle arrest, and inhibition of angiogenesis	Breast cancer	Phase 2	NCT00843167
			Melanoma	Phase 1	NCT01568996
Sulforadex (SFX -01)	Electrophilic modification of KEAP1 -Cys-151	It promotes programmed cell death/apoptosis, induces cell cycle arrest, inhibits angiogenesis, reduces inflammation, alters susceptibility to carcinogens, reduces invasion and metastasis, and exhibits antioxidant and anti-inflammatory properties	Breast neoplasm	Phase 1/2	NCT02970682
			Prostate cancer	Phase 1	NCT02055716
					NCT01948362
Curcumin (pro-oncogenic role)	Electrophilic modification of KEAP1 -Cys-151	The aberrant level of HO-1 promotes the Nrf2 downregulating genes, which results in the chemopreventive action and cancer promotion	Neoplasms	Phase 2	NCT02944578
			Prostate cancer	Phase 3	NCT01750359

### 9.1 Nuclear Factor Erythroid 2-Related Factor 2 Inhibitor

Nrf2 exhibits dual roles, such as being pro-oncogenic and anti-oncogenic. Hence, Nrf2 is involved in the inhibition of cancer development, and aberrant Nrf2 expression is also involved in cancer progression. In metastatic cancer, the contribution of Nrf2 to cancer progression could be minimized or eliminated by exploring some Nrf2 inhibitors. Vitamin C is a potent Nrf2 inhibitor and is used in the suppression of Nrf2 translocation (Mostafavi-Pour et al., 2017). Following the treatment of breast cancer cells with vitamin C, there was a substantial decrease in the expression of Nrf2 mRNA and protein levels. The nuclear/cytosolic Nrf2 ratio was lowered by 1.7-fold in MDA-MB-231 cells, 2-fold in MDA-MB 468 cells, 1.4-fold in MCF-7 cells, and 1.2-fold in A549 cells after treatment with vitamin C. In a dose-dependent manner, sequential treatment with vitamin C reduced endogenous ROS generation (*p* = 0.027). The findings suggested that vitamin C treatment could be developed as an adjuvant for cancer patients with Nrf2 overexpression (Mostafavi-Pour et al., 2017).

Brusatol, another class of Nrf2 inhibitors, diminishes the protein levels of Nrf2 in MDA-MB-231 breast tumor cell lines (Ren et al., 2011). In recent studies, it was observed that in mammospheres obtained from breast tumor cell lines, brusatol diminishes the protein levels of Nrf2 and deposition of intracellular ROS due to the increased cytotoxicity of Taxol (Yuan et al., 2017; Muralimanoharan et al., 2018). Berberine has recently been discovered to have anticancer properties in breast cancer by causing oxidative stress (Tang et al., 2009; Kim S. et al., 2012). Zhang and colleagues focused on BT-474 and AU-565 breast cancer cells that were resistant to lapatinib, a new tyrosine kinase inhibitor of HER2/EGFR (epidermal growth factor receptor) that was used in the study to treat breast cancer that is HER2-positive. Liquid nanocrystalline nanoparticles were developed to improve the solubility and anticancer properties of berberine in MCF-7 breast cancer cells (Zhang R. et al., 2016).

Parthenolide is a sesquiterpene class of Nrf2 inhibitor with antitumor and anti-inflammatory activities based on the control of reactive oxygen species (ROS) (Zunino et al., 2007; Mathema et al., 2012). By increasing Nrf2 downregulation and enhancing ROS generation, parthenolide (PN) and its soluble counterpart dimethyl amino parthenolide (DMPN) have been demonstrated to diminish mammosphere development in triple-negative breast cancer (TNBC) cell lines, as well as the survival of mammosphere-derived CSCs, most likely through increased ubiquitination and proteasome degradation (Carlisi et al., 2016). A combination of parthenolide and vinorelbine stealthy liposomes was developed for the suppression of breast cancer. Plumbagin, a member of the naphthoquinone class of Nrf2 inhibitors, is well known for its antitumor properties and redox impairment facilitated by plumbagin, which leads to ROS-dependent cell death in tumor cells. Loading of plumbagin in transferrin-bearing liposomes dramatically increased plumbagin uptake by tumor cells, resulting in improved anti-proliferative and antiapoptotic activity (Kapur et al., 2018).

### 9.2 Nuclear Factor Erythroid 2-Related Factor 2 Inducers

Activation of Nrf2 has primarily been reported as therapeutic, but a recent study has indicated that depending on the status of Nrf2 activation, the process can also be pro-oncogenic. According to a recent study, oncogenic signaling may influence Nrf2 activity by raising its mRNA levels. The oncogenic activation of K-RAS and B-RAF, depending on this, is sufficient to raise Nrf2 mRNA levels and enable ROS detoxification in human cancer cells. Curcumin, sulforaphane, and oltipraz, which have been identified as Nrf2 activators, have been discovered to be non–target-specific and may raise the risk of “off-target” toxicity due to their potential to interact with the cysteines of other enzymes and proteins. Molecular instability, decreased membrane permeability, and poor bioavailability of several Nrf2 modulators are also of significant concern (Telkoparan-Akillilar et al., 2021). Several Nrf2 inducers are used to elevate and translocate Nrf2 into the nucleus and activate the ARE mechanism for the detoxification of the cells.

## 10 Conclusion

Nrf2 plays a crucial role in cellular redox homeostasis in healthy as well as cancerous cells. In the absence of Nrf2, ROS production is upregulated, which leads to DNA damage and tumor development. Nrf2 is directly involved in managing the expression of GSH, TXN, and NADPH and controls the level of ROS. Interestingly, under oxidative stress, not only does Nrf2 regulate NADPH but ROS can also produce NADPH oxidase, which further activates Nrf2. These findings suggest the role of Nrf2 as an oncogenic factor. The variety of molecules that can be utilized to create better treatment options for breast cancer involve Nrf2-associated events. Therefore, a better understanding of cellular events and signaling cascades would enable finding a correct therapeutic regimen against breast cancer.
